# Effect of Storage Conditions and Storage Periods on Seed Germination in Eleven Populations of *Swertia chirayita*: A Critically Endangered Medicinal Herb in Himalaya

**DOI:** 10.1100/2012/128105

**Published:** 2012-04-19

**Authors:** Bharat K. Pradhan, Hemant K. Badola

**Affiliations:** Biodiversity Conservation and Management, G.B. Pant Institute of Himalayan Environment and Development, Sikkim Unit (Pangthang), P.O. Box 40, Gangtok, Sikkim 737 101, India

## Abstract

Effect of different storage conditions (room temperature, 4°C, and −15°C) and different storage periods over 24 months on seed germination in *Swertia chirayita* collected from different altitudes in Sikkim Himalaya was determined. Multivariate ANOVA revealed significant (*P* < 0.0001) effect of storage condition and storage period on seed germination and mean germination time. Seed germination percentage significantly (*P* < 0.01) varied between 87.78% (Sc5) and 100% (Sc2) during initial testing. Comparatively, high seed germination, low mean germination time, and low rate of fall in seed germination percentage in seeds stored at 4°C over different storage period were recorded. In addition, above 50% seed germination in majority of the populations even after 24 months of storage suggests 4°C as the most appropriate storage condition for long-term storage of seeds of *S. chirayita*.

## 1. Introduction

Storage of seeds as *ex situ *germplasm is an essential step for the long-term conservation of plant genetic resources. Maintaining seed viability for longer period is very essential to preserve the genetic integrity in stored samples. Since very early days, simple techniques have been adopted to maintain the seed viability in both domesticated and wild sources [1–8]. Inappropriate storage medium [9, 10] such as room temperature storage often results in low seed germination, seed deterioration, and loss of viability, which are natural phenomenon during storage [11, 12]. Several factors, namely, temperature, nature of the seeds, seed moisture content, relative humidity, and so forth, influence the seed longevity during storage [3, 4, 13–21]. There is a close relationship between the loss of seed viability during storage and the accumulation of genetic damage in the surviving seeds [22–24]. Seed moisture content, temperature, and storage periods are among the main factors affecting above relationship [25]. Slight increase in temperature and moisture may promote fungal growth [13] and insect development in seeds [26]. Depending on the duration and method adopted, drying and long-term storage may lead to considerable reduction in germination or to eventual death of the seeds. Before storage, if the seeds are not properly dried, the high moisture content may reduce the seed viability by promoting fungal growth. Such deterioration could further result in decline of seed germination capacity [27]. Proper storage conditions, however, may effectively retain substantial viability in seeds over a considerable storage period [4, 19, 20, 28]. Such approaches are especially crucial in case of endangered species, where judicial use of seeds as valuable genetic material through standardizing proper storage mechanism is a precondition to strengthen species conservation programme.

*Swertia chirayita* (Roxb. ex Fleming) H. Karst (Gentianaceae) is one of the highly marketed (nationally and internationally) critically endangered [29] medicinal herbs of Himalaya and prioritized at the top for the conservation through *ex situ* cultivation by an international experts' exercise [30]. As an erect, about 3–5 ft, biannual or triennial herb, S*. chirayita* is locally known as Chirowto or Pothi Chirowto or Kalo Chirowto and distributed in temperate Himalaya from Kashmir, Nepal, Bhutan along 1200–3000 m asl. The stems are robust, branching; the leaves are broadly lanceolate; the flowers occur in large panicles and are greenish yellow, tinged with purple; the capsules are egg shaped containing numerous minute seeds. In *S. chirayita*, both self-pollination [31] and cross-pollination [32] have been reported. The species bears huge pharmacological importance [33]; whole plant is used in traditional medicine; however the root is considered to be the most effective and bitter part. It has anti-inflammatory, hypoglycemic, antifungal, antibacterial, antimalarial and antioxidant properties, and considered to be a bitter tonic, febrifuge, and laxative and is used in fever, burning of body, intestinal worms, and skin diseases, and so forth [34]. *S. chirayita* is used in all kinds of fever in a variety of forms and in combination with other medicines. In Sikkim, decoction of plant obtained through boiling the entire plant is used to cure fever, cold, cough, diarrhea, and stomachache [35]. Also, *S. chirayita* is used in mental disorders, effective in curing gastric ulcers, liver diseases, and possess anticarcinogenic properties [34]. Further, the species is practiced in the preparation of herbal drug, such as Diabecon, D-400, and Himoliv.

Critically endangered standing of *S. chirayita* further necessitates its propagation and mass multiplication, for which a protocol targeting an appropriate and relatively longer period of storage of seeds would be vital and urgent need of the time. This will strengthen both *in situ* as well as *ex situ* conservation of the species. The present study was undertaken with an aim to test the effect of long-term storage of seeds under different storage conditions on seed germination in *S. chirayita*. The study was conducted on eleven populations from different parts of Sikkim Himalaya, India. These populations were different than those earlier tested for seed storage at 4°C, except Sc1 to Sc4, which were recollected from the same population sites [4]. Till present reporting, except our earlier work [4] there is no publication available on the effect of long-term storage on seed germination in *S. chirayita*, and first time covering different storage conditions.

## 2. Materials and Methods

### 2.1. Experimental Design

During November–December 2006, freshly ripened seeds of *Swertia chirayita* were collected from 11 populations located along 1600 m asl to 2700 m asl in different parts of Sikkim Himalaya, India (27°04′–28°07′ North and 88°00′–88°55′ East). Seeds from all populations were tested for three storage conditions over time. During seed collection, 15–20 plants/site were selected randomly from the middle of each population, to avoid edge effect, if any and not to disturb natural spread of the population, practicing sustainable harvesting. The seeds procured were pooled separately for each population and brought to the laboratory, cleaned thoroughly for impurities, and dried in room temperature for 15 days. Before further processing, all seeds for each population were mixed thoroughly to minimize effects of single source plant on germination

Seed moisture content was determined by oven drying (60°C; 48 hrs) 50 seeds in 3 replicates from each population. For each population 10 healthy fruits were considered for seed counting. Seed size was measured using 30 seeds per population under microscope (10 seeds each in 3 replicates). Seed viability test using 2,3,5, triphenyle tetrazolium chloride solution could not be conducted because of the minute seed size and the difficulty in finding the detached embryo [4]. For each population, immediately after room drying, seeds were tested for their initial germination potential. The remaining seed lots were stored in three different experimental conditions, namely, room temperature (25 ± 5°C), and in refrigerator at 4°C and at −15°C, in properly sealed specimen tubes. The seeds were periodically tested for their germination viability at six-month interval for next 24 months.

For each germination test, seeds were at first disinfected with sodium hypochlorite solution (4% w/v available chlorine) for 5 sec. to reduce the incidence of fungal attack. Disinfected seeds were washed thoroughly with double distilled water (DDW) and soaked in DDW for 24 hours. The soaked seeds were placed in Petri-plates (90 mm dia.) lined with single layer filter paper (Whatman No. 1) saturated with DDW. For each population, three replicates of 30 seeds each for each of three storage condition were used and Petri-plates then placed in a seed germination chamber at 25°C ± 2°C, with alternating light (14/10 hrs. photoperiod). Experiment was performed in a randomized design. The seeds were kept moist (using DDW) and checked every day. Visible protrusion of the radical was the criterion to score seed germination [4]. The germinated seeds were counted and removed. Seeds were observed daily until constant reading obtained. The germination experiment was observed up to 45 days in each of the testing at six-month interval.

### 2.2. Statistical Analysis

Univariate and multivariate ANOVA (analysis of variance) was performed using General Linear Model in SPSS 10.0 for windows (SPSS Inc. 1989) to determine the effect of populations, storage conditions, and storage period and their interaction on seed germination percent and mean germination time (MGT). MGT was calculated using the equation MGT = Σ(*nd*)/Σ*N*, where *n*—number of newly germinated seeds after each incubation period in days *d* and *N*—total number of seeds germinated at the end of the test [36]. Bonferroni test was employed to determine the variation in means of seed germination and MGT. The present experiment was designed to determine aging rates in seeds of *S. chirayita* stored under different storage conditions. Percent germination value obtained for the different populations prior to storage served as control value and a way to evaluate the variability in percent germination for periodical storage periods. By recording changes in the percentage germination and the time required for seeds to germinate, deterioration was evaluated. Pearson's correlation analyses were performed for examining the effect of altitude of the sites on seed size and on the seed germination.

## 3. Results

**Figure 1:**
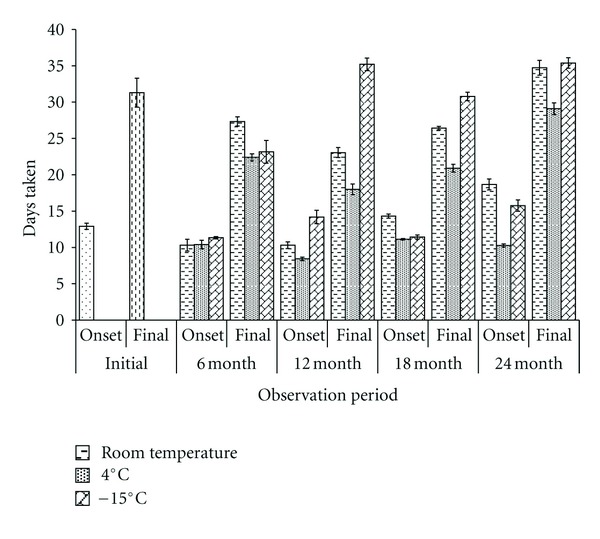
Storage effect on onset and final seed germination in *Swertia chirayita* (*vertical bars indicate the standard deviation*).

Populations of *Swertia chirayita* differed significantly for various seeds characteristics (Table 1). Altitude of the seed collection site showed insignificant negative correlation with seed sizes (seed length: *r* = −0.195; seed width: *r* = −0.082). Multivariate ANOVA revealed significant effect of population from different altitudes, different storage conditions, storage period, and their interaction on seed germination percentage and mean germination time (Table 2). Minimum 12 to 16 days (average: 13 days) was required for onset and 20 to 40 days (average: 31 days) for the completion of seed germination in most of the populations for all the three storage conditions and storage periods (Figure 1).

**Figure 2:**
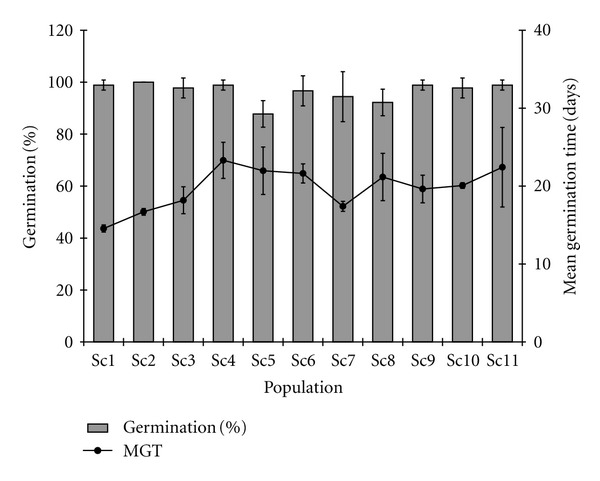
Mean seed germination and mean germination time (MGT in days) during initial testing in *Swertia chirayita* (*vertical bars indicate the standard deviation; 30 seeds* × *3 replicates per population used*).

**Figure 3:**
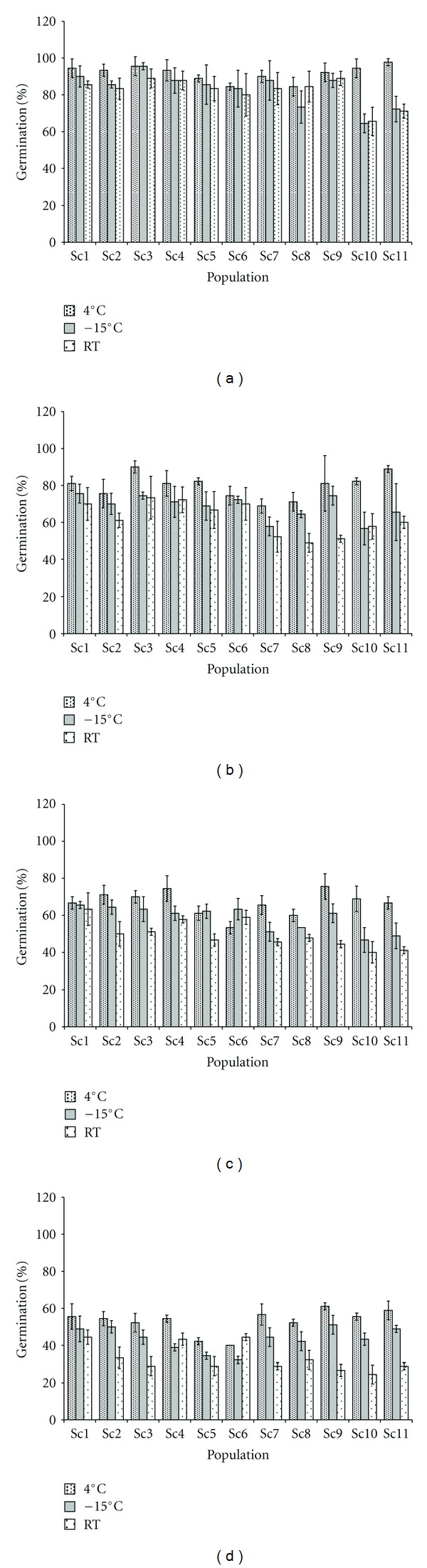
Effect of storage conditions and storage period on mean seed germination in populations of *Swertia chirayita* (a) 6 month; (b) 12 month; (c) 18 month; (d) 24 month (*vertical bars indicate the standard deviation; 30 seeds* × *3 replicates per population used; RT: room temperature*).

At the initial test, seed germination percent significantly (*P* < 0.01) ranged from 87.78% (Sc5) to 100% (Sc2) (Figure 2). For the seeds stored at 4°C, 73% (8 populations) of the total populations recorded over 90% seed germination after 6 months; 64% (7 populations) of the total populations recorded over 80% germination after 12 months; 91% (10 populations) of the total populations recorded over 60% germination after 18 months and 70% (8 populations) of the total populations recorded over 50% seed germination after 24 months of storage (Figures 3(a), 3(b), 3(c), and 3(d)).

For the seeds stored at −15°C, Sc3 recorded highest and Sc10 recorded the lowest germination after 6 months (Figure 3(a)); Sc1 and Sc10 recorded the highest and the lowest seed germination after 12 (Figure 3(b)) and 18 (Figure 3(c)) months of storage, respectively; however, after 24 months of storage, Sc2 recorded the highest and Sc6 recorded the lowest seed germination (Figure 3(b)).

Storage in room temperature resulted in above 80% seed germination in 9 (82% populations) of the total populations after 6 months. Here, the percent seed germination ranged between 48.89% (Sc9) and 73.33% (Sc3) after 12 months; 45% of the total populations recorded above 50% germination after 18 months, and the seed germination recorded below 33% in 82% of the total populations after 24 months of the storage (Figures 3(a), 3(b), 3(c), and 3(d)).

Collectively, significant reduction in germination percent was observed with the increasing storage duration compared to initial test (*P* < 0.0001) in all the three storage conditions in populations of *S. chirayita*. Cumulatively for all the populations, seeds stored at 4°C showed significantly (*P* < 0.0001) higher germination percent than −15°C and room temperature stored seeds. Similarly, the seed germination was significantly higher in −15°C stored seeds over room temperature stored seeds (*P* < 0.0001). The altitude of the seed collection site showed negative correlation with seed germination in seeds stored at room temperature at all the storage periods (12 month: *r* = −0.398; 18 month: *r* = −0.255; 24 month: *r* = −0.334).

**Figure 4:**
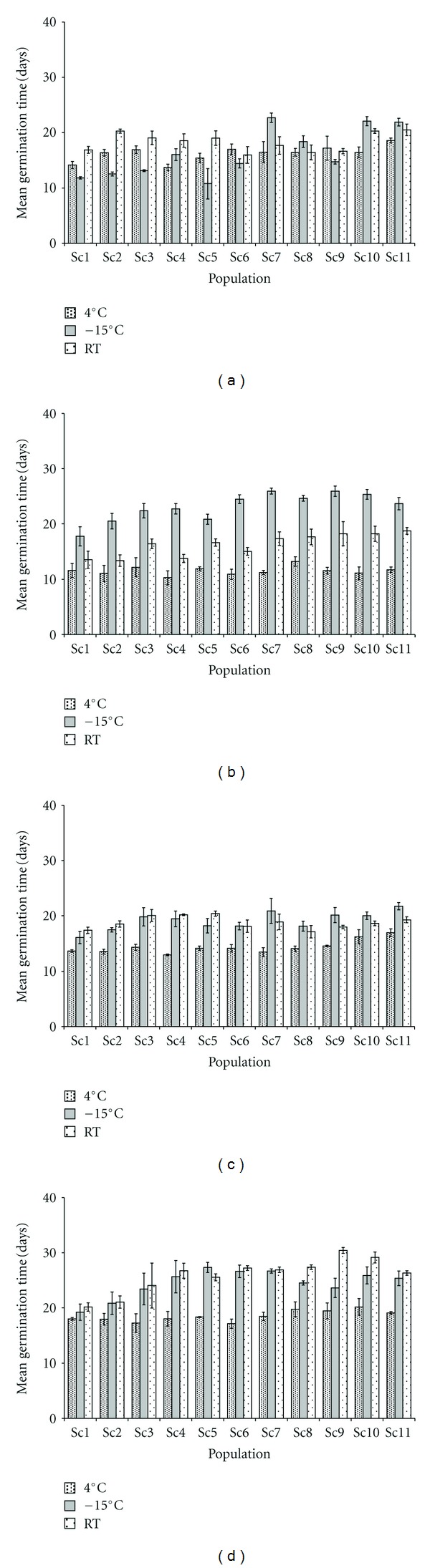
Effect of storage conditions and storage period on mean germination time in populations of *Swertia chirayita* (a) 6 month; (b) 12 month; (c) 18 month; (d) 24 month (*vertical bars indicate the standard deviation; 30 seeds* × *3 replicates per population used; RT: room temperature*).

Significant variation (*P* < 0.0001) in mean germination time (MGT) was observed amongst the storage conditions (Figures 4(a), 4(b), 4(c), and 4(d)) compared to the initial test (Figure 2). For example, seeds stored for 12 months at 4°C recorded the lowest MGT in all populations (Figure 4(b)); similarly, 64% of the total populations recorded lowest MGT after 6 months of storage at −15°C (Figure 4(a)) and 12 months of storage in room temperature (Figure 4(b)).

**Figure 5:**
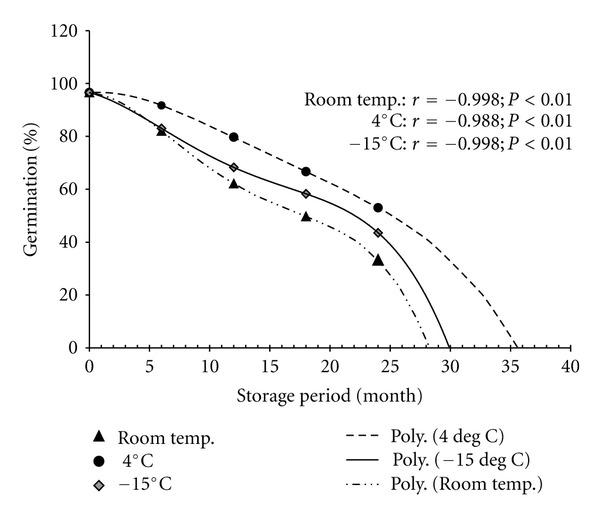
Seed viability (expressed as germination) as affected by storage period for different storage conditions in *Swertia chirayita *(*cumulative data from all populations*).

Significant fall (*P* < 0.01) in seed germination was observed in all the three storage conditions with the increase in storage time (Figure 5). The rate of fall in germination percent per 6 month was higher in room temperature (15.86%) followed by deep freeze (13.26%) and 4°C (10.89%) stored seeds. At this rate of fall, seed looses its viability completely by approximately 36 months in 4°C stored seeds which is slower compared to room temperature and deep freeze (Figure 5).

## 4. Discussion

The present study indicated that 19% to 44% seed moisture content after drying can retain seed viability under different storage condition for longer period in *S. chirayita*. This is supported by the study of Hampton and Hill [37] who stated that 10% to 40% seed moisture is desirable for retaining good seed longevity. In our earlier study [4], 16% to 43% seed moisture resulted in 100% seed germination during the initial testing and above 50% seed germination in majority of population in the same species up to 18 months of storage. However, McCormack [38] stated that higher seed moisture (greater than 18%) results in loss of seed viability and vigour. Bhatt et al. [39] recorded the low seed germination with the moisture content of 22% to 29% in freshly collected seeds of *Swertia angustifolia*. In another study, even after maintaining the moisture content of 15% to 21% in the domesticated seeds of *S. chirayita*, the present authors observed low germination at the initial testing [40]. This indicates source-specific requirement of seeds for desirable moisture content for long-term viability.

Our present investigation, testing seeds for long-term storage, confirms that the storage temperature/condition significantly affects the seed germination capacity as indicated by Bradbeer [17]; nonetheless, it varies greatly by species and storage conditions [41]. Our study indicates that the seed deterioration rates may vary depending on the storage conditions, and the germination percent and/or seed viability gradually declines with increase in storage period as reported by Yilmaz and Aksoy [42], irrespective of different storing conditions; nevertheless, Wang et al. [43] reported an increase in seed germination with the increase in duration of seed storage time in some subalpine species. The reported variability in percent seed germination for the seeds collected from different populations at the same time or from the same population at different times even if they are provided with the same treatments or test conditions [44, 45] is not applicable to *S. chirayita* in Sikkim Himalaya. Our present study with *S. chirayita* revealed that the seeds collected from the same location for two different years in case of four populations, that is, Sc1, Sc2, Sc3, and Sc4, after a year gap resulted in 98% to 100% germination (roughly the similar trend) in comparison to the earlier result of 100% germination for the same populations [4]. This is an indication that the initial germination potential may remain similar over different times if right collection strategy is maintained, especially the time of collection and the selection of same spots in natural habitats.

In our present study, six-month storage did not show much variation in percent germination among the storage conditions suggesting the suitability of all three conditions for short-term storage, which is often practiced in several Himalayan herbs [19, 20, 46]. For 4°C storage, all the populations showed higher percent germination after 12 months, comparing to −15°C storage. In general, above 60%, 70%, and 80% germination after 12-month storage in room temperature, at −15°C, and at 4°C, respectively, suggests that up to 12 months, the seed storage in room temperature can also be opted provided due care be taken in respect of moisture content level prior to storage. After 18 months, abrupt fall in the percent seed germination below 48% in 55% populations indicates the room temperature as inappropriate condition of storing seeds for longer period in* S. chirayita*. Seed storage at −15°C maintained above 60% germination with majority of the populations (7 populations) after 18 months of storage. Continued storage for 24 months at −15°C resulted in decrease in the seed germination (below 50%) for nine populations, which suggests this as unsuitable condition for the long-term seed storage.

Comparatively, the seeds stored at 4°C resulted in higher percent germination than the seeds stored at room temperature and at −15°C, on subsequent testing. For all three storage conditions, progressive and significant reductions in percent seed germination were observed with increasing duration of the storage. On an average basis, loss of seed viability was much faster at room temperature followed by −15°C stored seeds, but gradual at 4°C suggesting it as the most appropriate condition for the long-term storage of *S. chirayita *seeds. Chauhan and Nautiyal [47] reported much faster loss of seed viability at room temperature (10–35°C) and retaining of seed viability for more than two years (storage at 0–4°C in refrigerator) in *Nardostachys jatamansi*. Onyekwelua and Fayose [3] stated that the seeds cannot be stored at subzero temperature probably due to freezing injury resulting from ice formation, which can alternatively be controlled by placing them in airtight sealed containers. Our present finding suggests that storing seeds of *S. chirayita* at −15°C can be a second option after 4°C; however, here the seeds lose their viability early compared to 4°C even if they are placed in air tight containers. Many studies reported that the seed storage at 4°C was effective for germination after 6 months [19] to 12 months [4, 28]. In several other species, loss of seed viability is observed within a few months of storage at room temperature [48, 49]. Seed moisture content is adjusted as per the relative humidity of the surrounding air which changes with the air temperature and seeds differ in the way they adjust their moisture content to humidity. In refrigerator (4°C), both temperature and the relative humidity are properly maintained thereby retaining the seed viability for longer period, relatively.

For *Swertia chirayita*, in our earlier study [4], an increasing trend in MGT was recorded after six months of storage at 4°C, on subsequent testing for 18 months. Whereas, in our current study with four retested populations (Sc1 to Sc4) in second year and the remaining sets of populations, the case little differed as MGT declined till 12 months in majority of the populations, and then MGT enhanced subsequently when provided with similar storage condition. Similar trend in MGT reduction was observed for the seeds stored in room temperature. For the seeds of *Kochia prostrata* stored in laboratory and under shed, mean germination time decreased as storage time increased, and varied unpredictably for cold room-stored seeds, and remained unchanged for the freezer-stored seeds [50], is not applicable to *S. chirayita*. Nonuniformity in MGT observed in the seeds on subsequent tests is rather confusing in the case of −15°C stored seeds in *S. chirayita*. However for the seeds stored in room temperature and at 4°C, the MGT first decreased up to 12 months of storage compared to initial test, which increased with the prolonged storage period.

## 5. Conclusion

Our present study stressed on the high seed germination potential as well as variability in seed germination in eleven populations of *S. chirayita* collected from different altitudes in Sikkim Himalaya as in our earlier study [4]. It concludes convincingly that the storage condition highly affects the seed germination percentage in *S. chirayita*, which declines with increasing storage duration irrespective of storage condition. The study indicated that the storage at 4°C temperature can retain seed viability for the longer period than other storage conditions and suggests it as the best/effective storage condition for the seeds of *S. chirayita*. However, the room temperature and −15°C storage can be opted for short-term storage up to one year in *S. chirayita*. Equally important is the recommendation on practicing sustainable harvesting taking care of precise time and exact spot in the site, where earlier collections were made using potential populations for conservation programmes in case of endangered species.

## Figures and Tables

**Table 1:** General features and seed characteristics of different population of *Swertia chirayita* collected in Sikkim.

Name of populations	Altitude m asl	No. of seeds/fruit (10 fruits/population for seed counting)	Seed weight (mg) 50 seeds × 3 replicates/population	Seed length (*μ*m) 30 seeds (10 × 3 replicates)	Seed width (*μ*m) 30 seeds (10 × 3 replicates)	Moisture content (%) 50 seeds × 3 replicates/population	Habitat
Sc1—Luing (ES)	2126	209 ± 54.69	1.5 ± 0.06	486 ± 5.80	355 ± 12.29	25.8 ± 12.10	Ridge, moist grassy slope with *Cryptomeria japonica* and *Castanopsis tribuloides. *
Sc2—Railgaon (ES)	1948	297 ± 76.97	1.6 ± 0.06	532 ± 13.94	383 ± 22.68	22.4 ± 3.27	Shrubberies with *C. japonica* and *C. tribuloides. *
Sc3—Upper Pangthang (ES)	2176	309 ± 56.27	1.6 ± 0.10	502 ± 28.33	380 ± 11.55	29.1 ± 2.31	Shrubberies, moist grassy slope with *Quercus lamellosa* and *C. tribuloides. *
Sc4—Jaunbari (SS)	1651	222 ± 46.34	1.2 ± 0.10	454 ± 14.22	374 ± 12.00	39.1 ± 6.08	Moist grassy slope with *Alnus nepalensis* and *Michelia lanuginosa. *
Sc5—Tiffin dara 2 (SS)	1744	269 ± 25.25	1.4 ± 0.06	406 ± 8.05	362 ± 15.63	26.7 ± 7.82	Moist grassy slope with *Cryptomeria japonica* and *Alnus nepalensis. *
Sc6—Dhupi Dara (ES)	2124	258 ± 29.01	1.8 ± 0.12	418 ± 13.11	378 ± 16.97	18.8 ± 1.97	Forest-Shrubberies with *Cryptomeria japonica. *
Sc7—Deewani Taar (WS)	2055	294 ± 35.91	1.2 ± 0.10	358 ± 10.44	325 ± 10.69	36.5 ± 7.84	Forest-Shrubberies with the dominance of *Alnus nepalensis. *
Sc8—Gumpa Dara (WS)	1987	260 ± 31.58	1.2 ± 0.06	372 ± 4.16	351 ± 4.58	29.7 ± 4.27	Forest-shrubberies with *Cryptomeria japonica and Alnus nepalensis. *
Sc9—Hilley (WS)	2697	260 ± 33.55	1.4 ± 0.12	389 ± 9.54	369 ± 10.00	44.3 ± 3.71	Forest-Shrubberies with *Lithocarpus pachyphylla *and *Arundinaria maling. *
Sc10—Tendong (SS)	2099	230 ± 33.07	1.6 ± 0.10	390 ± 10.97	372 ± 7.51	27.0 ± 2.23	Forest-Shrubberies with *Castanopsis tribuloides* and *Quercus* sp as dominant.
Sc11—Ravangla (SS)	2160	256 ± 23.06	1.3 ± 0.00	320 ± 6.03	297 ± 3.21	25.6 ± 8.88	Mixed forest of *Castanopsis tribuloides, Quercus lamellosa, Machilus edulis,* and *Cinnamomum tamala. *

LSD (*P* < 0.05)		38.49	0.14	21.53	21.14	10.54	

*F* value		5.23	15.95	76.93	12.94	4.23	

ES: East Sikkim; WS: West Sikkim; NS: North Sikkim; SS: South Sikkim; ± denotes the standard deviation.

**Table 2:** Result of multivariate ANOVA, showing the effect of populations, storage conditions, and storage periods and their interaction on seed germination and mean germination time in *Swertia chirayita* (using General Linear Model in SPSS 10.0 for windows; SPSS Inc. 1989).

Source	*F*	Sig.	*F*	Sig.
	Germination (%)		Mean germination time	
Population (P)	14.443	.000	42.392	.000
Storage condition (SC)	279.387	.000	716.784	.000
Storage period (SP)	1038.901	.000	519.862	.000
P × SC	8.914	.000	9.144	.000
P × SP	3.993	.000	5.728	.000
SC × SP	6.789	.000	147.827	.000
P × SC × SP	1.810	.001	4.878	.000
